# Surgical Techniques for Managing Complex Distal Radius Fractures: A Technical Report

**DOI:** 10.7759/cureus.107139

**Published:** 2026-04-16

**Authors:** Angelos Assiotis, Adam Rumian, Nikolaos P Sachinis, Harpal S Uppal

**Affiliations:** 1 Department of Trauma and Orthopaedics, Lister Hospital, Stevenage, GBR; 2 First Orthopaedic Department, Aristotle University of Thessaloniki, Thessaloniki, GRC; 3 Department of Trauma and Orthopaedics, General Hospital "Georgios Papanikolaou", Thessaloniki, GRC

**Keywords:** distal end radius fracture, distal radius fracture management, trauma and orthopedics, upper extremity trauma, volar locking plate

## Abstract

Fractures of the distal radius are common injuries and may result from high-energy or low-energy trauma. Treatment options include non-surgical and surgical management, and although high-level evidence does not support surgical management for all of these fractures, there is a clear increasing trend in plate fixation for fractures of the distal radius.

Where there is an indication to manage these surgically, open reduction and internal fixation of distal radius fractures is a core surgical skill that most trauma surgeons possess. However, some of these fractures may present in complex or atypical patterns. We present our experience with such cases and a stepwise surgical technical note with several tips on managing specific components of these complex fractures.

## Introduction

Distal radius fractures are common presentations in trauma surgery, as they account for approximately 18% of all fractures [1]. Although a large proportion of these may be treated non-surgically, there are specific indications for surgical management. Intra-articular fracture lines and significant fragment displacement are often indications for surgical management, and such fractures may be broadly treated with closed reduction and percutaneous pin stabilisation, open reduction and internal fixation, or ligamentotaxis, either with an external fixator or a spanning plate.

When considering surgical fixation with volar locking plates, the most commonly used implants in open reduction and internal fixation of these fractures, we note that there is an abundance of available implants on the market, and these are implanted through a volar approach centred around the flexor carpi radialis (FCR) tendon. The surgical steps for stabilising most of these fractures are widely known by surgeons who deal with such injuries, and we do not aim to describe the management of routine distal radius fractures. Rather, we would like to present our experience with complex or severely displaced articular fractures of the distal radius, where the well-known steps in surgical management are likely to result in suboptimal surgical outcomes, and where a more nuanced approach may benefit patients and surgeons. We illustrate this as a technical report through several cases that we have treated at our institution in the past several years.

In the context of preoperative planning of distal radius fractures in our unit, we use computerised tomography (CT) scans in any presentation of a displaced articular distal radius fracture or a fracture that presents with significant comminution of the meta-diaphysis of the radius. We feel that preoperative CT scans allow for accurate surgical planning that is otherwise not entirely feasible with plain radiographs [2].

Although the steps we present in this paper are certainly not required for the vast majority of these fractures, surgeons who regularly deal with such injuries could benefit from using them if the need arises.

## Technical report

We present several surgical tips and steps that may be used in managing specific technical challenges in complex distal radius fractures. In each section, where possible, we have included preoperative and postoperative radiographs and CT scans that demonstrate the application of these tips in clinical practice. All radiographs and CT images are anonymised.

Extended FCR approach

We routinely use the extended FCR approach as described and popularised by Orbay et al. [3]. This approach reliably allows for full visualisation and instrumentation of the volar lunate fragment and adequate mobilisation of the fracture fragments, especially when operating on this more than two weeks after the injury. The technique to manage this involves the release of the radial septum, as described by Orbay et al. [3], followed by the introduction of a blunt instrument, such as a MacDonald dissector, into the dorsal fracture site, allowing elevation of the extensor compartments and dorsal callus from the fracture site and therefore facilitating fracture mobilisation and reduction. We would use the next step, which is pronation of the radial shaft and complete visualisation of the fracture fragments, in cases of articular intercalary rotated fragments that require direct handling and reduction, otherwise not achievable through a traditional volar approach.

Reduction technique

**Figure 1:**
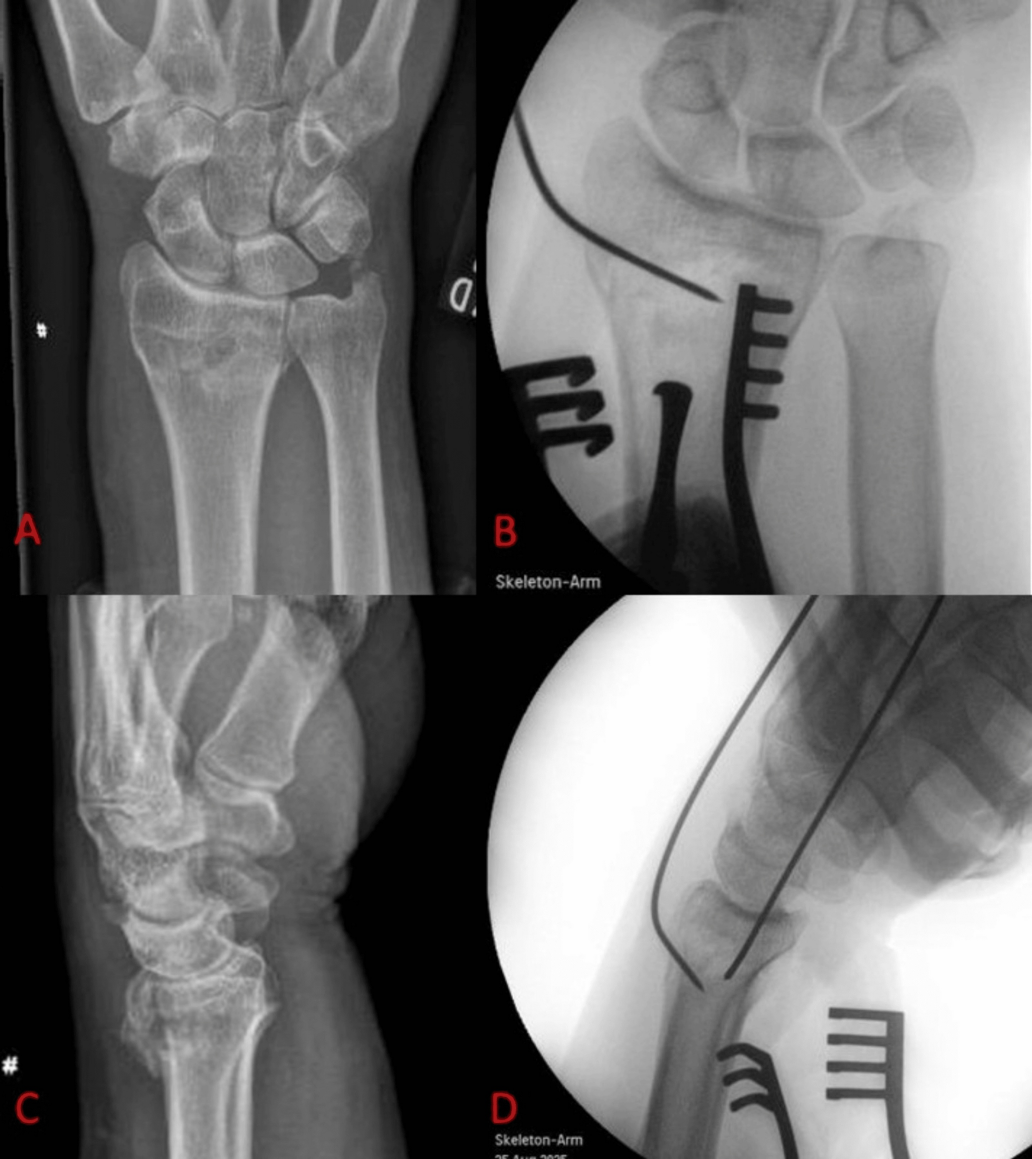
Radiographs demonstrating distal radius fracture reduction technique. (A) Anteroposterior radiograph of a distal radius fracture, demonstrating loss of radial height and inclination. (B) Intraoperative lateral radiograph showing a 1.6 mm wire correcting the coronal plane deformity. (C) Lateral radiograph of fracture, demonstrating dorsal tilt of distal fragment. (D) Intraoperative lateral radiograph showing second, dorsal 1.6 mm wire, resulting in complete fracture reduction.

Our research group has already published on the technique we routinely use to achieve temporary reduction of distal radius fractures, without the need for an assistant [4]. The use of 1.6 mm K-wires, as described in our published technique (Figure 1), allows for near-anatomical reduction in the majority of distal radius fractures, and allows the surgeon to focus on the appropriate handling and placement of the volar locking plate in order to achieve definitive fixation.

**Figure 2:**
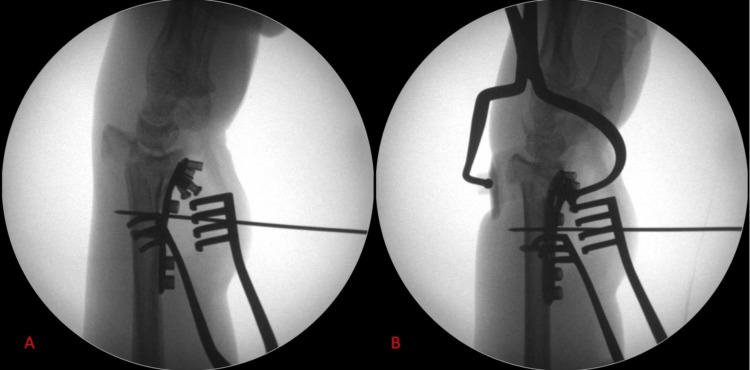
Example of the use of the soft tissue bone reduction clamp (A) Intraoperative lateral radiograph showing displacement of dorsal fragment (B) After application of the reduction clamp, there is near-complete reduction of the dorsal fragment.

In cases where the wire reduction technique does not fully correct the radial volar tilt, we resort to the use of the soft tissue bone reduction clamp manufactured by DePuy Synthes (Warsaw, USA). This instrument reliably fully corrects the volar tilt, something that has been recognised in the past as a technical challenge in dealing with these fractures [5]. Due to its radiolucent dorsal reduction attachment, it may be left in situ while the plate is secured on the radius, without impeding intraoperative radiographs, as seen in Figure 2. 

Restoration of radial height

A common deformity in comminuted fractures of the distal radius is loss of radial height and secondary ulnar-carpal abutment. It is sometimes challenging to fully correct the radial height with longitudinal traction by the surgical assistant, especially when these fractures are operated on more than two weeks after the date of injury. In our practice, the deforming force of the brachioradialis tendon on the radial styloid is dealt with either by performing a step-cut in the tendon or by elevating the tendon from the radial styloid before attempting correction of radial height. After reducing the fracture in all planes, if there is residual loss of radial height, a volar locking plate is applied to the shaft, with a cortical screw in an oblong screw hole (most modern volar locking plates have this option available). We would then carry out the distal fixation of the epiphyseal fragments before releasing the shaft screw and pushing the entire plate/epiphysis construct distally in order to restore radial height. While maintaining height reduction, the surgical assistant will then re-tighten the cortical screw on the shaft, thereby securing the radial height. A further point that we would like to make here is that we routinely use smooth locking pegs for the entire distal row, with the exception of the radial styloid. The rationale is that there is often significant comminution in the dorsal aspect of the distal radius, and the presence of threads in the distal pegs does not convey any biomechanical benefit to the resulting construct. Furthermore, the lack of threads to the distal row pegs makes their introduction more time-efficient. As mentioned earlier, the exception to this practice is the radial styloid, where we use threaded pegs/screws. The rationale for this is the fact that the radial styloid is often not as comminuted as the rest of the distal radial epiphysis and metaphysis, and the presence of threaded pegs allows for further stabilisation of this, often relatively intact, fragment.

Shaft plate fixation in osteoporotic bone

**Figure 3:**
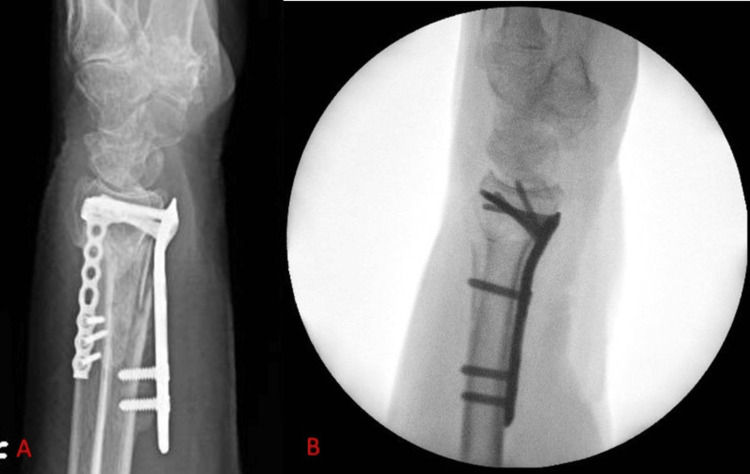
Lateral radiographs from two different cases, showcasing the importance of adequate shaft fixation in osteoporotic bone. (A) Lateral postoperative radiograph, demonstrating loss of fixation of the volar locking plate on the distal radius. Note that the plate is fixed to the shaft with only two screws. (B) Lateral postoperative radiograph demonstrating appropriate shaft fixation with three locking screws. Note that the non-locking slot has been left empty as the non-locking screw did not fully engage the far cortex due to comminution. Despite this, the three remaining locking slots on the plate allowed for adequate fixation to the shaft.

Osteoporotic fractures of the distal radius usually occur in elderly patients, with a tendency to affect elderly female patients. When securing the plate to the shaft with the first non-locking cortical screw, the plate may not be securely fixed. As we move on to deal with the epiphysis, shaft fixation may loosen or fail, resulting in unnoticed rotation of the plate in the coronal plane in relation to the shaft and the epiphysis of the distal radius. In order to manage this technical challenge, a suggested technique is to use a K-wire through the dedicated wire hole on the plate shaft (most modern volar locking plates share this feature) as soon as the cortical non-locking screw is inserted. This secures the plate position on the shaft, even if the cortical screw does not entirely secure the plate on the shaft of the distal radius. In managing these patients, a four-hole plate may be beneficial as opposed to a three-hole option, as this ensures that even if the non-locking cortical screw does not offer adequate stability when inserted, there are still three locking screw options for the shaft available, as shown in Figure 3.

Fracture distraction

**Figure 4:**
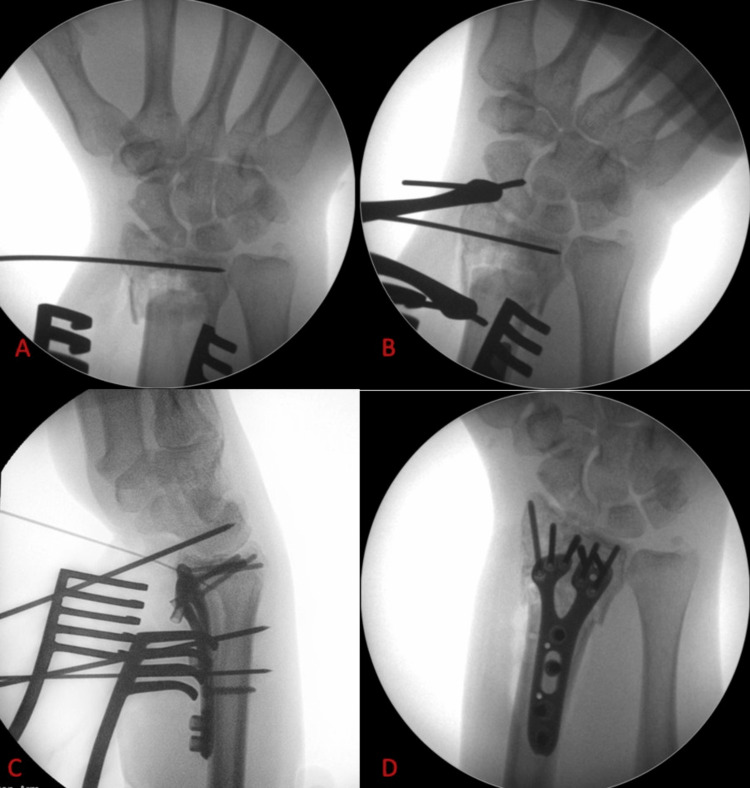
Example of fracture distraction, aiding fracture reduction. (A) Intraoperative radiograph showing loss of height and inclination of comminuted fracture of the distal radius. (B) Intraoperative anteroposterior radiograph showing near-complete correction of the deformity with the application of appropriate ligamentotaxis through a Hintermann distractor, with 1.6 mm K-wires in the radial shaft and the scaphoid. (C) Intraoperative lateral radiograph showing appropriate wire position in scaphoid and radial shaft, and satisfactory reduction of fracture. (D) Intraoperative anteroposterior radiograph demonstrating appropriate reduction of fracture.

In cases with significant metaphyseal and epiphyseal comminution of the distal radius, a technical challenge that sometimes has to be managed is the difficulty in restoring the radial height, with the methods mentioned in the point above this one. In such cases, the use of a distraction device is beneficial, as it allows for sustained and controlled distraction that a surgical assistant may not be able to deliver. In our practice, we use a Hintermann distractor, which is routinely used in foot and ankle surgery. Depending on which column of the wrist we plan to distract (between the radial and intermediate columns), strategic placement of 1.6 mm or 2 mm K-wires in the carpal bones (the scaphoid for the radial column and the lunate for the intermediate column) and in the radial shaft allow for significant distraction to be applied, which can be maintained until definitive stabilisation is achieved with a volar locking plate. Such an example is presented in Figure 4. Surgeons should carefully plan the position of these wires, especially the one that is inserted in the radial shaft, such that it does not prevent the appropriate placement of the volar locking plate.

Coronal deformity correction 

**Figure 5:**
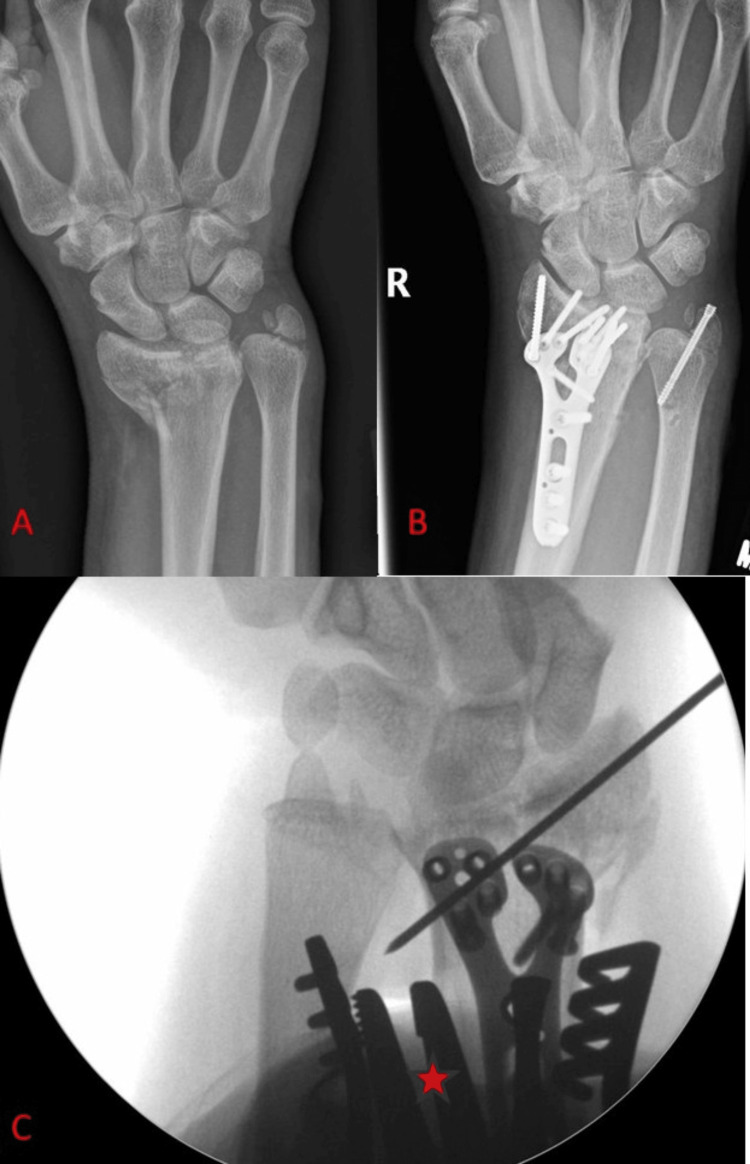
Case example where a laminar spreader is placed between the radial and ulnar shafts. (A) Anteroposterior radiograph showing significant displacement and comminution of the distal radius fracture. Note the collapse of the lateral column, resulting in radial tilt of the distal fragment. (B) Anteroposterior radiograph showing the near-complete correction of the previous deformity after the intraoperative use of the laminar spreader. (C) Intraoperative radiograph of a different patient, showing appropriate placement of the blades of the laminar spreader between the distal radial and ulnar shafts, noted with a red asterisk.

In cases with significant radial column comminution and/or bone loss, there is often a coronal plane deformity where the distal radial epiphysis translates radially in relevance to the shaft. Reducing the epiphysis onto the radial metaphysis is often challenging in such cases and not easily achieved by simple ulnar deviation of the hand by the assistant. In such cases, a small laminar spreader is very useful when placed between the distal radial and distal ulnar shafts and engaged in that position. This results in relative distraction between the radial and ulnar shafts, and as the distal radioulnar joint is competent in the vast majority of these cases, this distraction results in reduction of the distal radial epiphysis over the radial metaphysis, as seen in Figure 5. In other words, the distal ulna ‘pulls’ the distal radius epiphysis with it and places it back onto the distal radial metaphysis.

Forearm position for distal row fixation

After provisional reduction, as presented above, and application of the plate to the shaft, attention is then turned to the distal fixation of the articular fragments to the plate. Although the order of fixation of the articular fragments could be debated, we propose that this step is performed with the forearm in mid-prone position and the surgeon inserting the distal row screws/pegs in this position. There are two benefits in this position: first, it allows for prompt intraoperative lateral radiographs to ensure that the distal pegs are appropriately placed in relation to the subchondral plate. The second, and most important benefit, is that with the forearm in the mid-prone position, the distal radius epiphysis remains appropriately reduced onto the volar locking plate; this is relevant as several patients present without complete and free external rotation in their shoulders and as such, when stabilising the epiphysis with the forearm on the operating table (and thus, in a supinated position), there is a tendency for the surgical assistant (who usually controls the patient’s hand) to supinate the hand and distal fragment, ‘away’ from the shaft, which tends to pronate due to the unimpeded pull of the pronator muscles. This becomes evident when the distal fragment is sometimes stabilised with a gap in relation to the volar plate and may be associated with flexor tendon impingement on the metalwork, especially of the flexor pollicis longus tendon. Stabilising the distal fragments with the forearm in mid-prone position mitigates against this technical error.

Reduction of volar rim fragments

**Figure 6:**
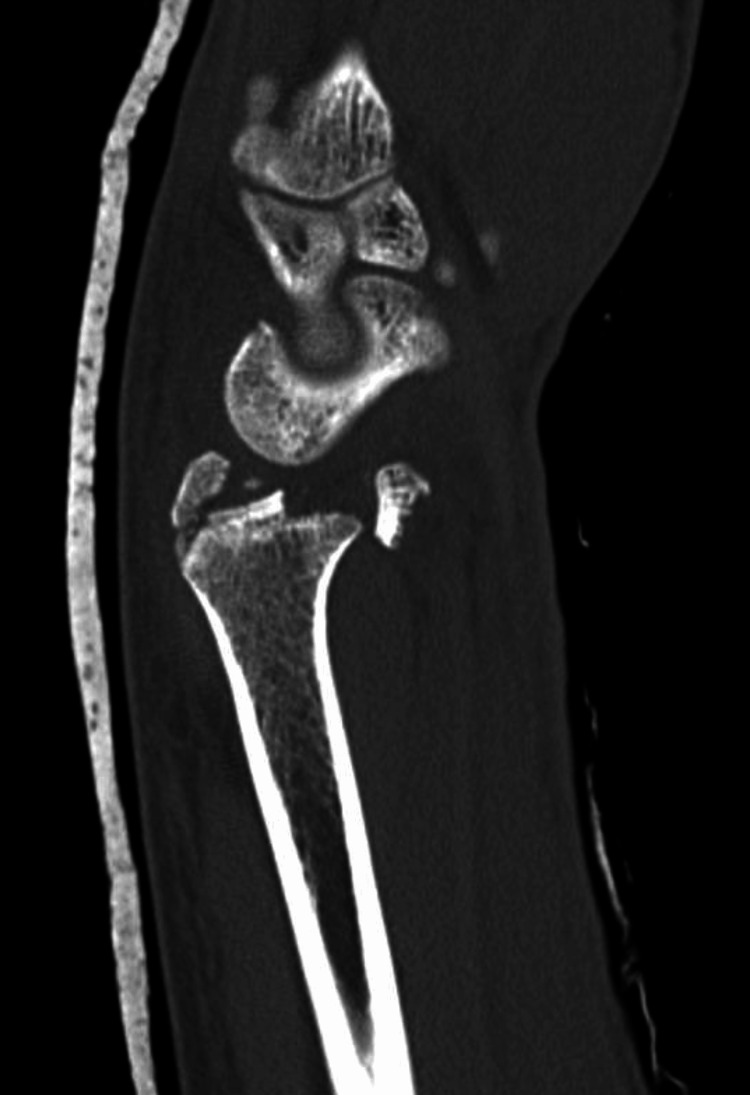
Case example of patient with volar rim fragment that may not be amenable to standard volar plate fixation in isolation. Sagittal CT reconstruction showing complete extrusion of articular fragment of scaphoid fossa in a volar direction.

Volar rim fragments often involve the volar lunate fragment, although they sometimes also involve the scaphoid fossa, especially in high-energy injuries, such as the one seen in Figure 6, where a young male patient presented with a high-energy fracture/dislocation of the distal radius.

**Figure 7:**
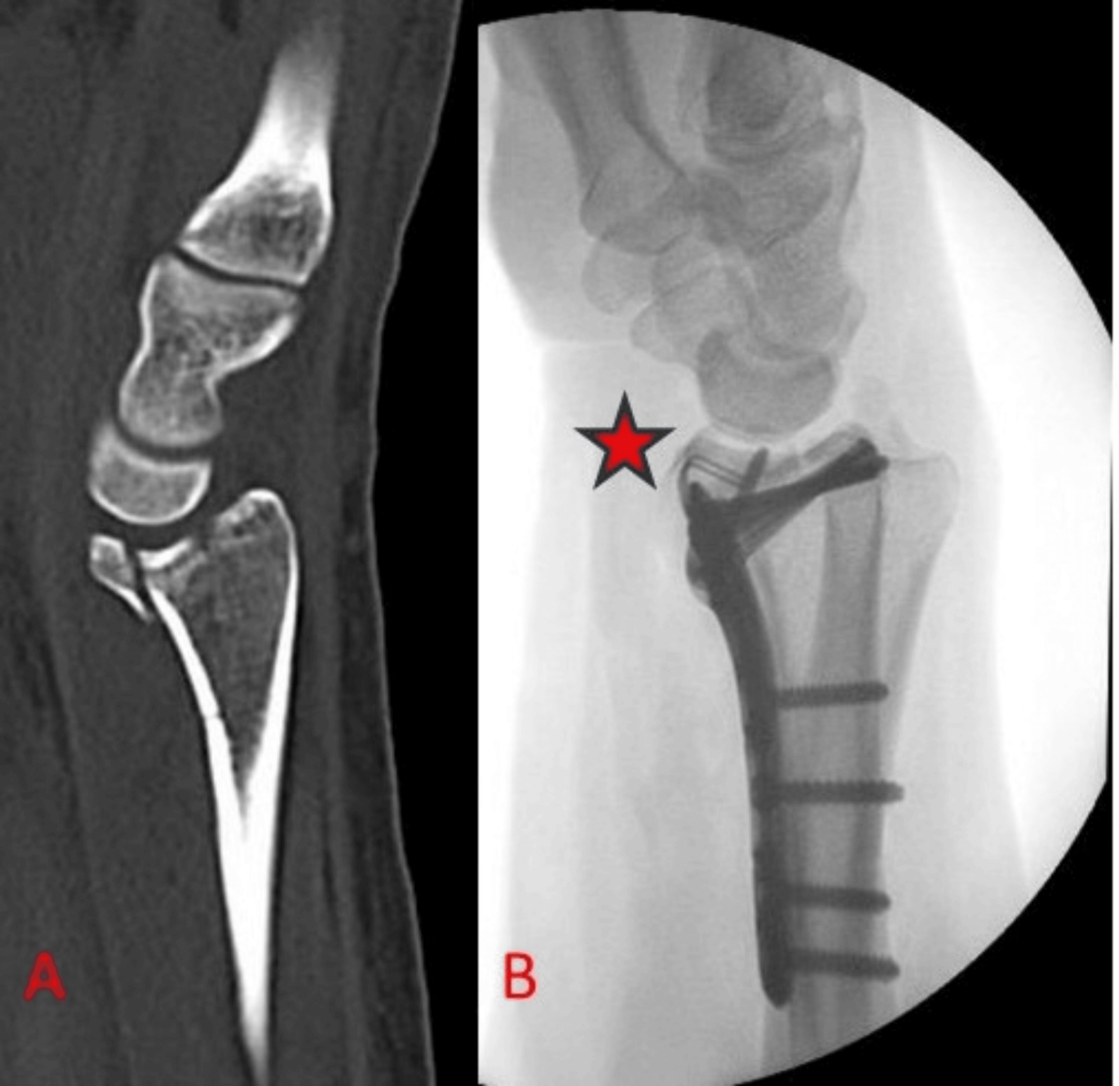
Case example of stabilisation of volar rim fragment (A) Preoperative sagittal CT reconstruction showing a very distal volar rim fragment of the lunate fossa and resulting radiocarpal instability. (B) Intraoperative lateral radiograph showing appropriate placement of hook attachment (noted with red asterisk) to volar locking plate, securing the volar rim fragment.

In such cases, even the use of modern volar locking plates, which allow placement of the implant just proximal to the watershed line, is often not enough to stabilise these rim fragments, which may lead to secondary loss of reduction due to the radiocarpal ligaments, which attach to them. In these rare presentations, either hook plate attachments (if the system in use offers that option), such as in Figure 7, or fine wires, which are used to stabilise these fragments to the intact radial metaphysis, allow for appropriate stabilisation.

If wires are used, they need to be meticulously trimmed and ideally placed under the volar locking plate in order to reduce the incidence of wires backing out and the subsequent reoperation to remove them.

Intercalary fragment reduction

**Figure 8:**
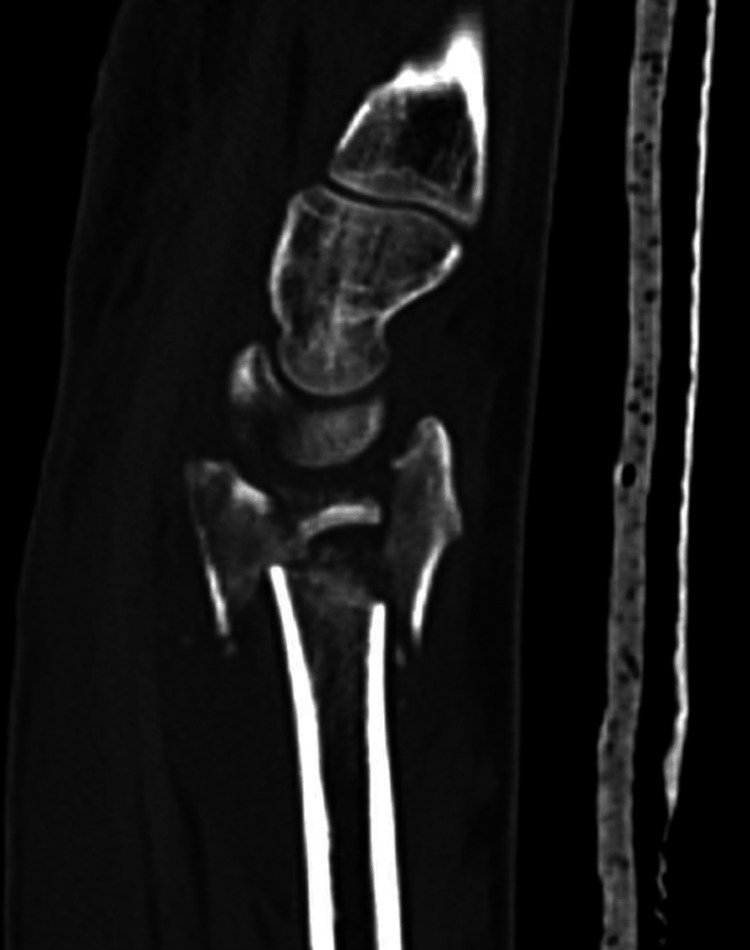
Sagittal CT reconstruction showing lunate fossa intercalary fragment rotated by 180 degrees.

Die punch and intercalary fragments of the distal radius are often associated with inadequate reduction and fixation, as they are not often readily accessible from a volar or dorsal surgical approach (Figure 8).

**Figure 9:**
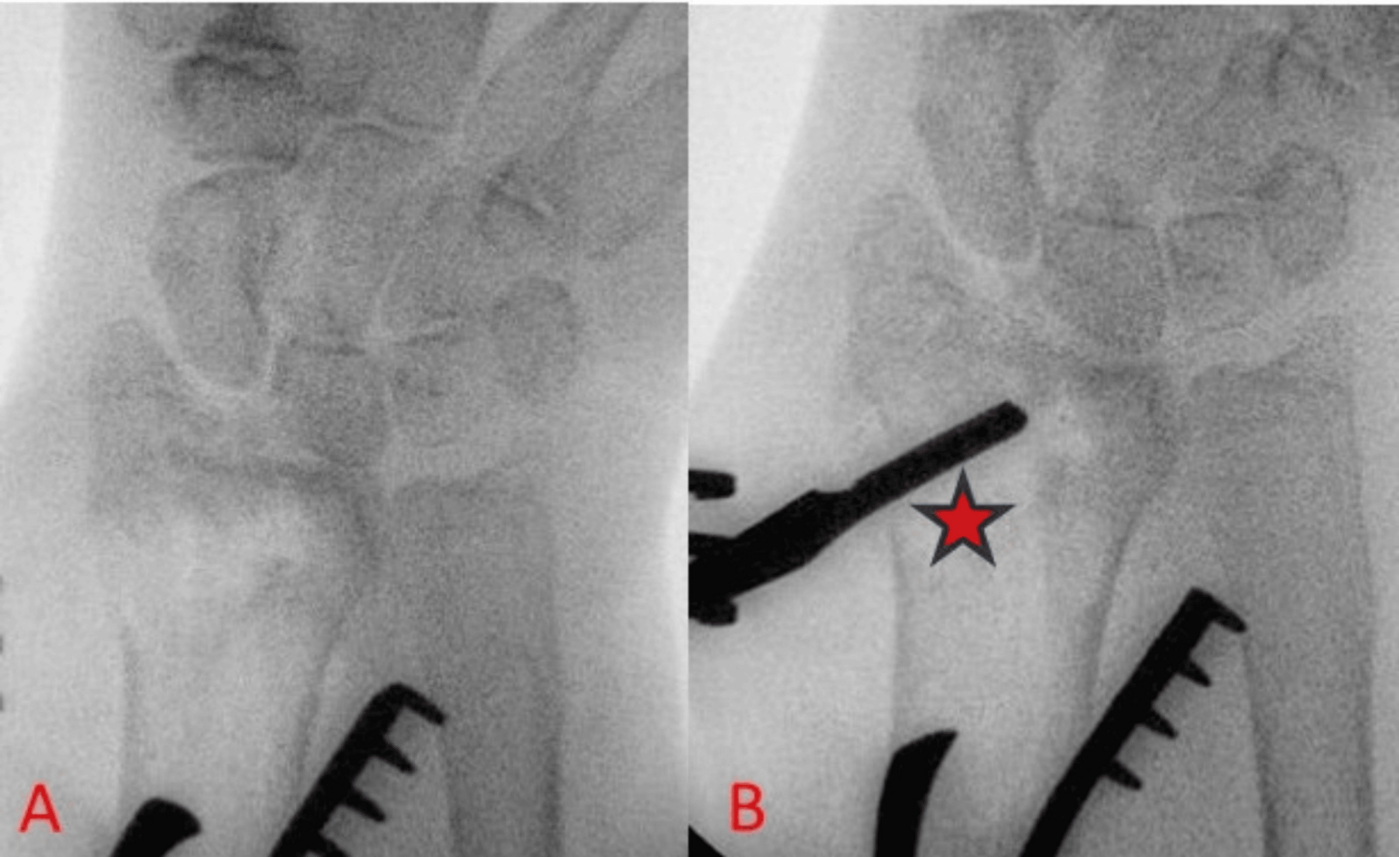
Reduction technique for die-punch fragments (A) Intraoperative anteroposterior radiograph showing a displaced die-punch fragment of the distal radius. (B) Intraoperative radiograph showing the introduction of a dissector (noted with red asterisk) through the fracture window, elevating the die-punch fragment in line with the rest of the articular surface.

A helpful technique includes the image-guided introduction of a Watson Cheyne or MacDonald dissector through the existing volar fracture line, which is then used to reduce the die-punch fragment, as seen in Figure 9.

**Figure 10:**
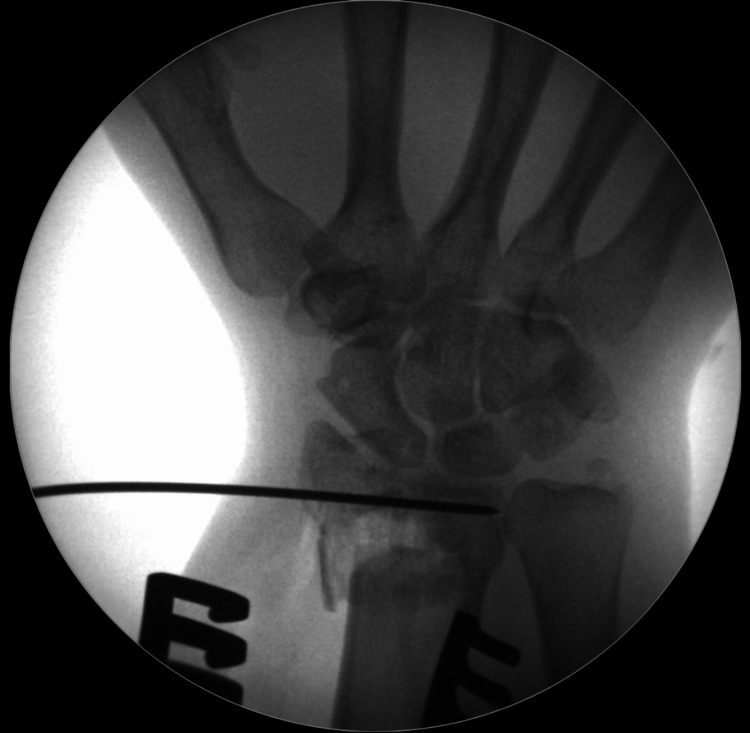
Once the die-punch fragment is elevated, it can be held reduced with a 1.2 mm wire inserted in subchondral bone from a radial to ulnar direction.

Reduction is then maintained with a 1.2 mm K-wire, which is introduced from a radial-to-ulnar direction, in the subchondral bone (Figure 10).

Use of this technique requires a preoperative CT scan, as the placement of the dissector and the wire, requires correct appreciation of the location and size of the die-punch fragment.

Concomitant volar and dorsal plate fixation

**Figure 11:**
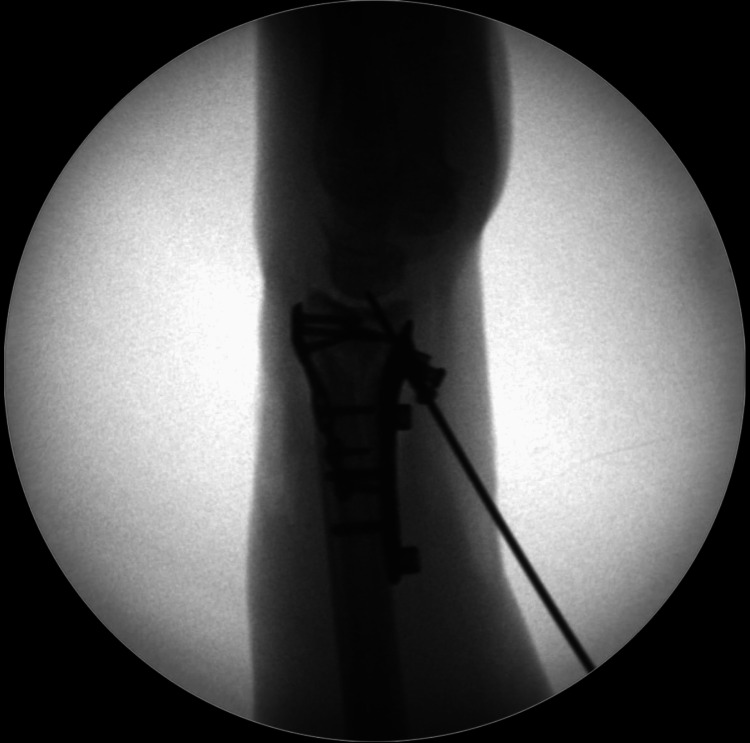
Lateral intraoperative radiograph of combined volar and dorsal plate fixation Although rarely required, a combined collar and dorsal approach and fixation is required. Here, the dorsal fragment would not fully reduce with closed methods, as there was incarceration of an extensor tendon between the dorsal fragment and the distal radius.

Modern volar locking plate evolution and current designs allow for distal/subchondral fixation of articular fragments and also for the use of long distal pegs/screws, and we believe that the use of dorsal plates for the distal radius is less commonly indicated nowadays. There are cases, however, where a combined dorsal and volar approach and fixation is indicated, such as when there is significant residual displacement of large dorsal fragments, even after the use of the aforementioned reduction techniques. This could be the case where there is incarceration of an extensor tendon in the fracture site, such as seen in Figure 11, where open reduction and fixation of the large dorsal lunate fragment was necessary, after the fifth extensor compartment tendon was released from its incarcerated position, volar to the dorsal distal radius fragment.

Dorsal tangential view

**Figure 12:**
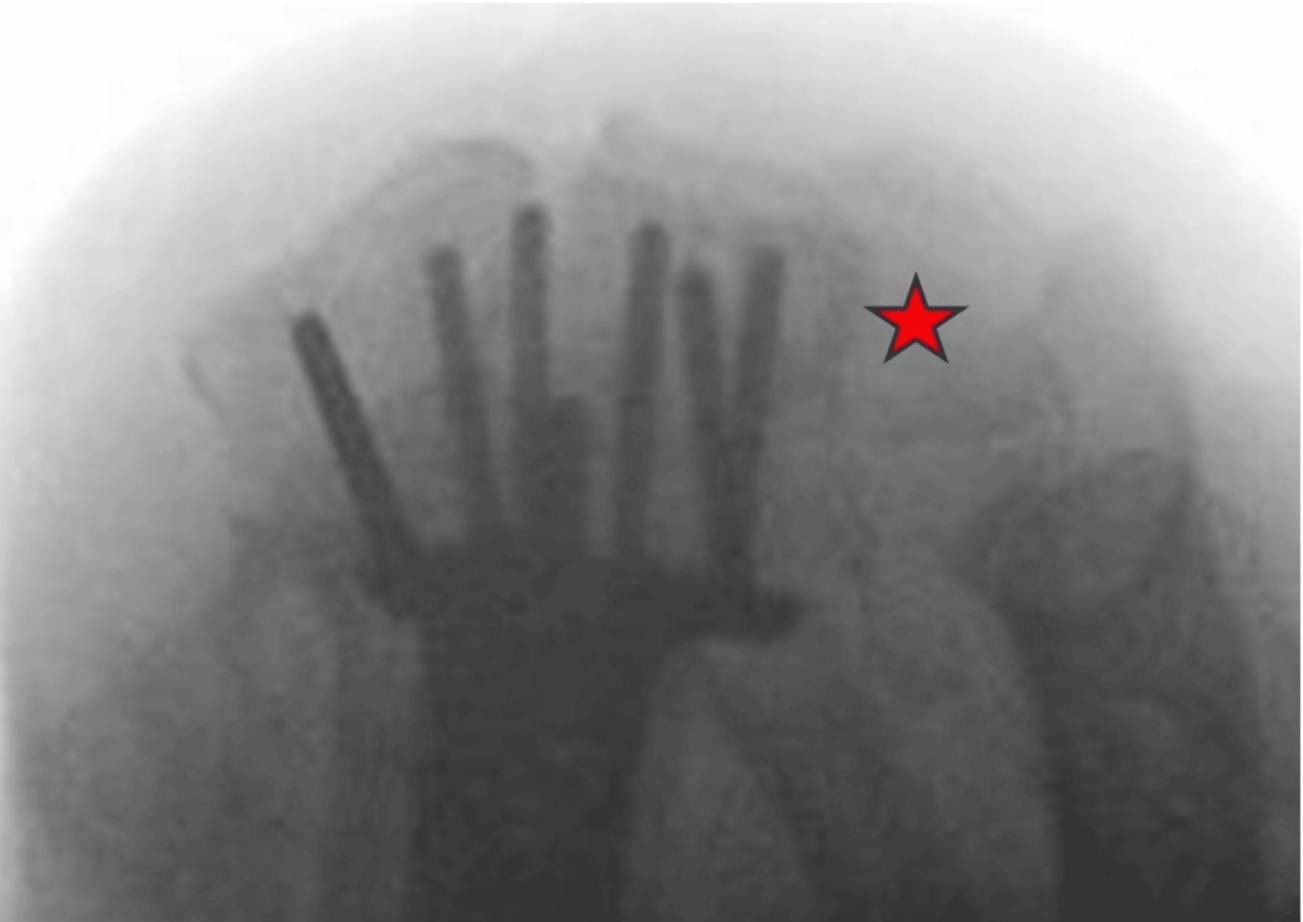
Skyline intraoperative radiograph after volar plate application This shows appropriate length of the distal locking pegs. Also, the sigmoid notch of the distal radius is clearly visible (noted with a red asterisk) and seen to be free from screw penetration.

When obtaining final intraoperative radiographs after the application of a volar locking plate, we support the routine use of the dorsal tangential view [6] or skyline view of the distal radius. This radiograph allows appreciation of two important parameters: first, it allows accurate visualisation of the tips of the distal row pegs/screws to ensure that these do not penetrate the dorsal cortex of the distal radius and therefore avoid impingement of the metalwork on the extensor compartment tendons. The second benefit of this view, as seen in Figure 12, is the accurate visualisation of the sigmoid notch of the distal radius and the ability to ensure that there is no screw penetration into the notch and, therefore, into the distal radioulnar joint.

Wrist spanning implants

**Figure 13:**
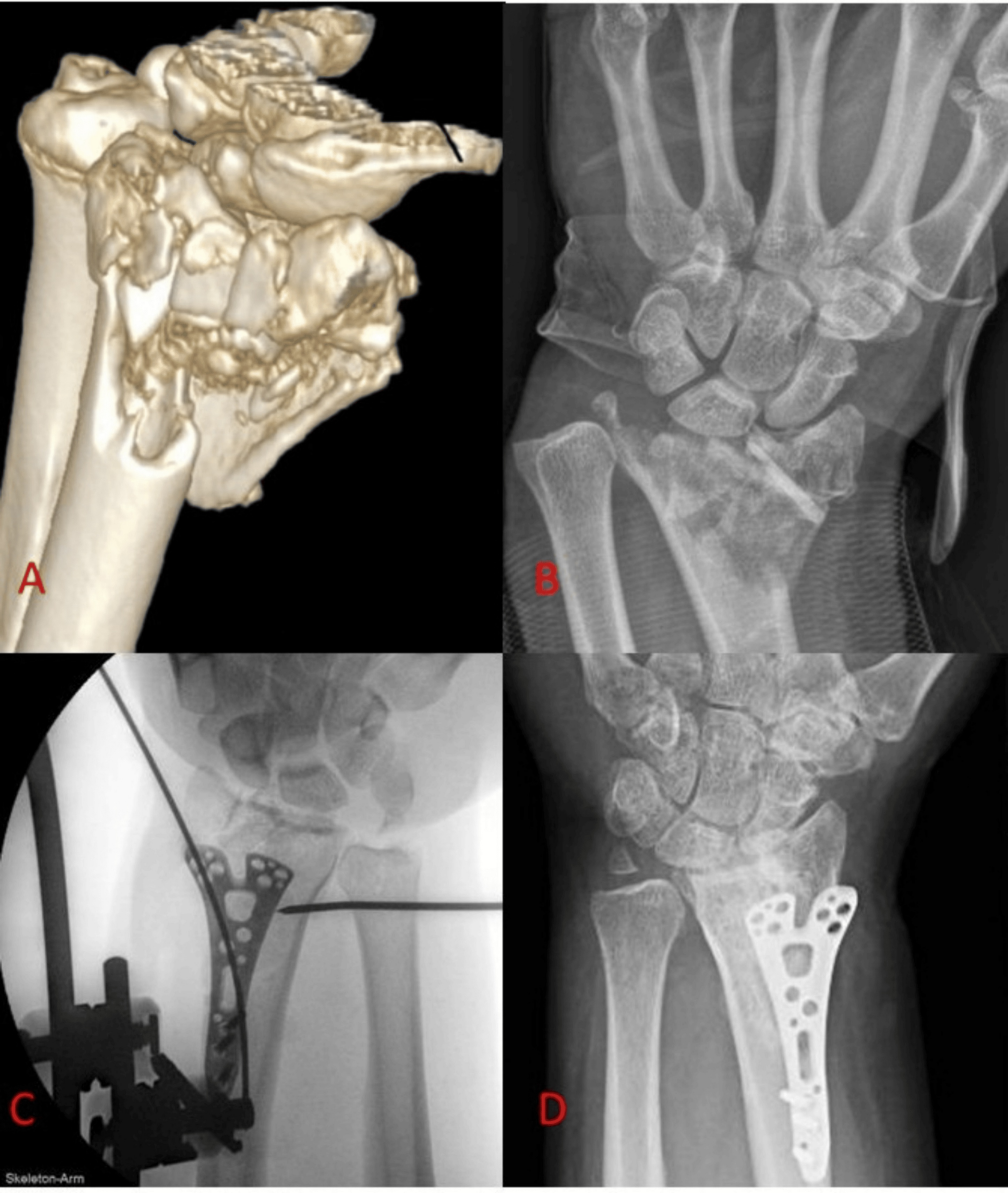
Case example of complex distal radius fracture, which was treated with combined external and internal fixation. (A) CT reconstruction of a highly comminuted fracture of the distal radius, not amenable to volar locking plate fixation. (B) Preoperative anteroposterior radiograph of the same fracture, which was subsequently stabilised with a combination of suture fixation, external fixation, and volar plate fixation. (C) Intraoperative anteroposterior radiograph demonstrating restoration of radial height with external fixator. (D) Postoperative anteroposterior radiograph demonstrating fracture union. The volar plate was removed subsequently.

Modern designs of volar locking plates offer the ability to manage more complex injuries than previous designs, as they have become more low-profile and may be placed more distally, adjacent to the watershed line of the distal radius. In our practice, the use of such implants has reduced the need to resort to implants that work through ligamentotaxis, such as external fixators or dorsal spanning plates. That said, we still sometimes manage fractures of such complexity (Figure 13), where having the option of either of the aforementioned implants is required and indicated.

When an external fixator or a dorsal spanning plate is implanted, appropriate preoperative counselling should take place because even after such implants are removed, patients often have to recover from significant postoperative wrist stiffness. Although it could be suggested that this significant stiffness could be related to the high-energy injury that led to the need to use a spanning implant in the first place, as opposed to a volar locking plate, this patient cohort should be consulted carefully regarding wrist stiffness after surgery.

## Discussion

Fractures of the distal radius are prevalent in children and adults, and their management may be non-surgical or surgical. Surgical management includes options such as wire fixation, plate fixation, spanning fixation with external fixators or spanning plates, and, more recently, wrist hemiarthroplasty [7,8]. With advances in the design and manufacturing of volar locking plates, there has been a clear increasing trend in managing these fractures with open reduction and internal fixation with a plate [9]. This trend is not fully supported by large randomised controlled trials, such as the DRAFFT trial [10] and the WRIST trial [11], where patient-reported outcome measures do not seem to be significantly different between different treatment modalities when distal radius fractures are assessed as a whole. In keeping with the findings of these studies, Nordback et al. suggested that the management approach should be to default to non-operative management in patients with reduced functional demands and to consider surgery in the form of corrective osteotomy if patients present with symptoms after fracture union [12].

There are still several fracture configurations for which surgical management should be offered to patients as the optimal modality. Such fractures include fracture/dislocations of the radiocarpal articulations, articular fractures with significant displacement and articular or non-articular fractures with significant shortening, or those which result in significant midcarpal adaptive instability. Such injuries are often best treated with surgical stabilisation. When deciding to manage these with volar locking plates, there is an abundance of implant options on the market, and as technology and manufacturing continue to evolve, these options allow for the management of injuries that previously would likely result in suboptimal functional outcomes. In other words, as these implants become lower-profile and have improved screw trajectories, they may be placed more distally and are more anatomical than previous versions of volar locking plates.

In recent years, there has been increasing appreciation and application of the column concept, in which the distal radius and ulna are divided into the radial, intermediate, and medial columns. Treatment strategies may be planned and, in a way, personalised, depending on which column is involved in the fracture presentation [2]. At the same time, there is a more nuanced understanding of the rationale behind the common fracture patterns that we observe in most patients. It has been demonstrated that articular fracture lines in the distal radius seem to present between ligament attachment areas [13]. It also seems that bone density is relatively higher adjacent to the radioulnar and radiocarpal joints [14], something that explains, to an extent, the fact that these areas are perhaps less often comminuted, compared with the rest of the distal radius. Relevant to this, fracture mapping studies [15] have demonstrated that the radial styloid is usually less comminuted than other areas of the distal radius and that there seems to be significant comminution dorsally, with often a small dorsal medial fragment. More than just offering insight into the pathoanatomy of these injuries, these studies allow for surgical planning to be carried out based on real anatomical considerations, rather than one of several older classification systems, none of which seem to be ideal.

The majority of distal radius fractures are approached through an FCR-centred approach, which comes in several variations and is often described as a ‘modified’ Henry approach. Although such an approach is adequate for the majority of these injuries, some fractures require improved exposure, and other variations, such as the extended FCR approach [3], become more useful.

Although several surgical techniques and modifications of these have been described in the past, to our knowledge, no comprehensive technical guide has been published on how to tackle specific surgical challenges that these fractures sometimes present with. Regardless of fracture type, the surgical objectives of stabilising fractures of the distal radius have not changed and include adequate reduction of the articular block onto the distal radial metaphysis and adequate union of these two main components [7]. In achieving this, our proposed surgical steps/tips are by no means exhaustive for all possible fracture configurations, but our experience with these has demonstrated that they are very useful in successfully managing the overwhelming majority of complex distal radius fractures.

## Conclusions

Although most distal radius fractures can be managed successfully with well-established techniques, we describe additional surgical tips and steps that may be used alone or in combination, as required, in order to manage specific technical challenges that sometimes present during the surgical fixation of complex fractures of the distal radius. In our paper, we do not present functional outcomes of a case series with the use of these techniques, as they only represent our institution's experience with specific challenging cases; therefore, these techniques should be adopted by the treating surgeon on a case-by-case basis.

We have employed these techniques over several years in our unit and believe that they can greatly assist surgeons in managing this pathology, where conventional reduction/stabilisation manoeuvres fail to yield an acceptable reduction.
